# Suppression of eukaryotic initiation factor 4E prevents chemotherapy-induced alopecia

**DOI:** 10.1186/2050-6511-14-58

**Published:** 2013-11-13

**Authors:** Zeina Nasr, Lukas E Dow, Marilene Paquet, Jennifer Chu, Kontham Ravindar, Ragam Somaiah, Pierre Deslongchamps, John A Porco Jr, Scott W Lowe, Jerry Pelletier

**Affiliations:** 1 Departments of Biochemistry, McGill University, Montreal, Quebec H3G 1Y6, Canada; 2 Memorial Sloan-Kettering Cancer Center, New York, USA; 3 Département de Pathologie et de Microbiologie, Faculté de Médecine Vétérinaire, Université de Montréal, Saint-Hyacinthe, Québec J2S 2 M2, Canada; 4 Howard Hughes Medical Institute, New York, NY 10065, USA; 5 Départment de Chimie, Université Laval, Ste-Foy, Quebec G1V 0A6, Canada; 6 Center for Methodology and Library Development, Boston University, 590 Commonwealth Ave., Boston, MA 02215, USA; 7 Department of Oncology, McGill University, Montreal, Quebec H3G 1Y6, Canada; 8 The Rosalind and Morris Goodman Cancer Research Center, McGill University, Montreal, Quebec H3G 1Y6, Canada

**Keywords:** Chemotherapy-induced alopecia, eIF4E, eIF4A, Translation initiation, Genetic engineered mouse model, Cyclophosphamide

## Abstract

**Background:**

Chemotherapy-induced hair loss (alopecia) (CIA) is one of the most feared side effects of chemotherapy among cancer patients. There is currently no pharmacological approach to minimize CIA, although one strategy that has been proposed involves protecting normal cells from chemotherapy by transiently inducing cell cycle arrest. Proof-of-concept for this approach, known as cyclotherapy, has been demonstrated in cell culture settings.

**Methods:**

The eukaryotic initiation factor (eIF) 4E is a cap binding protein that stimulates ribosome recruitment to mRNA templates during the initiation phase of translation. Suppression of eIF4E is known to induce cell cycle arrest. Using a novel inducible and reversible transgenic mouse model that enables RNA_i_-mediated suppression of eIF4E *in vivo,* we assessed the consequences of temporal eIF4E suppression on CIA.

**Results:**

Our results demonstrate that transient inhibition of eIF4E protects against cyclophosphamide-induced alopecia at the organismal level. At the cellular level, this protection is associated with an accumulation of cells in G1, reduced apoptotic indices, and was phenocopied using small molecule inhibitors targeting the process of translation initiation.

**Conclusions:**

Our data provide a rationale for exploring suppression of translation initiation as an approach to prevent or minimize cyclophosphamide-induced alopecia.

## Background

Chemotherapy-induced hair loss (alopecia) is an unmet challenge in clinical oncology and considered one of the most psychologically negative factors in cancer patient care. The psychological impact of chemotherapy-induced alopecia (CIA) is significant. In conjunction with vomiting and nausea, it is among the most feared side-effects of chemotherapy [1]. CIA is seen with alkylating agents (e.g., cyclophosphamide), cytotoxics (e.g., doxorubicin), antimicrotubules (e.g., paclitaxel), and topoisomerase inhibitors (e.g., etoposide) and is a consequence of perturbations of hair-follicle cycling and hair shaft production. No reliable preventative pharmacological approach for CIA is currently available [2].

Strategies aimed at protecting normal cells from chemotherapeutic agents may offer benefit to prevent CIA. One approach, known as cyclotherapy, aims to selectively and transiently induce cell cycle arrest in normal cells [3,4]. In proof of principle experiments, the MDM2 antagonist, nutlin-3a, was used to activate p53 and induce a reversible cell-cycle arrest in non-transformed cells - protecting them from S or mitotic phase inhibitors. In contrast, p53^-/-^ tumor cells do not cell cycle arrest and remain susceptible to chemotherapy [5-8]. However, nutlin-3a is not clinically approved, has poor efficacy *in vivo,* requires a high working concentration (200 mg/kg) in mice [9,10], and induces cell cycle arrest within a narrow concentration window (between 2 μM and 10 μM) [11,12]. There is thus a need to identify and test additional small molecules that could be used to entice a cyclotherapy response.

In eukaryotes, suppression of eukaryotic initiation factor (eIF) 4E activity slows G1 progression in yeast [13] and non-transformed mammalian cells [14,15]. eIF4E is required for ribosome recruitment during translation initiation and is thought to function through eIF4F, a heterotrimeric complex that consists of (i) eIF4E, a cap-binding protein; (ii) eIF4A, an RNA helicase required for generating a ribosome landing pad; and (iii) eIF4G, a large scaffolding protein [16]. Assembly of eIF4F is regulated by mTOR and is thought to be a nodal point mediating proliferative and survival consequences of increased signaling flux through the PI3K/mTOR pathway [17]. There is thus significant interest in identifying specific inhibitors of eIF4F for assessment as anti-neoplastic agents [17].

We have recently described the development of a novel inducible RNAi platform in the mouse that combines GFP-coupled shRNA technology with a Flp/FRT recombinase-mediated cassette exchange (RMCE) strategy to generate mice that conditionally express shRNAs [14,18]. Two strains that we generated enabled inducible and reversible suppression of eIF4E at the organismal level - the effects of which are well tolerated in the mouse [14,19]. One tissue in which this system shows high eIF4E suppression is in the skin, including hair follicle cells (this study). We therefore envisioned that this model would be useful for assessing a potential role for eIF4E suppression in CIA. Using a well-established protocol for studying CIA in mice [20], we demonstrate that transient eIF4E suppression prior to chemotherapy protects from CIA by decreasing apoptosis of hair follicle cells. These results provide genetic validation for targeting eIF4E as a mean to reduce CIA.

## Methods

### General reagents

Doxycycline hydrochloride (Sigma-Aldrich) was dissolved in water at 1 mg/ml with 5% sucrose and supplied to mice in their drinking water. Cyclophosphamide (Sigma-Aldrich) was resuspended in water and stored at 4°C. Nutlin-3a, paclitaxel, nocodazole, and vinorelbine were purchased from Sigma-Aldrich, resuspended in DMSO and stored at -20°C.

### Cell lines

Normal human primary fibroblast BJ/TERT (obtained from Dr. Joe Teodoro, McGill University) and MRC5 lung fibroblast cells (ATCC) were cultured in Dulbecco’s modified Eagle’s medium. All media was supplemented with 10% Fetal Bovine Serum (FBS), 100 U/ml penicillin/streptomycin (P/S), and 100 U/ml L-Glutamine. Cells were grown at 37°C and 5% CO_2_.

### Targeting construct and ES cell generation

**Figure 1:**
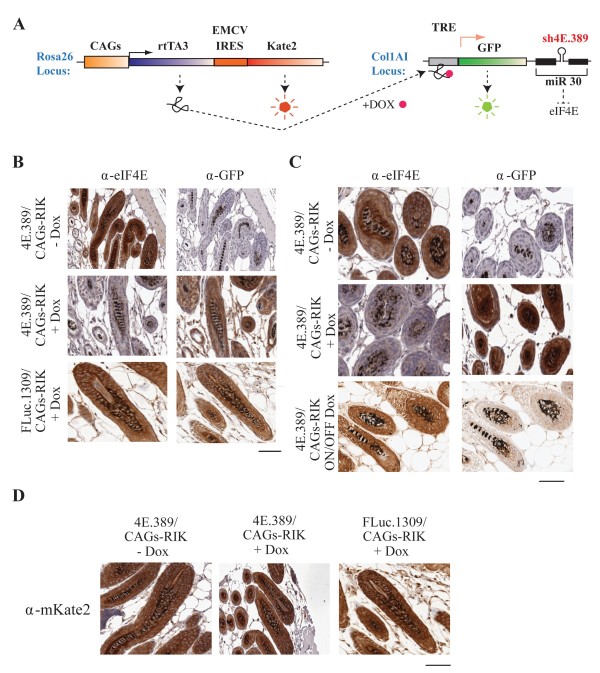
**Inducible and reversible suppression of eIF4E in hair follicle cells. (A)** Allele configuration at *Rosa26* and *Col1A*1 and loci of shRNA/CAGs-RIK mice designed to exhibit inducible and reversible expression of shRNAs. **(B)** Representative immunohistochemistry staining showing eIF4E and GFP staining in the hair follicles of vehicle-treated (-Dox) and Dox-treated (+Dox) (5 days) mice. Bar represents 50 μm. **(C)** Representative immunohistochemistry staining showing eIF4E and GFP staining in the hair follicles of vehicle-treated (-Dox) 4E.389/CAGs-RIK mice, doxycycline-treated (+Dox) (5 days) 4E.389/CAGs-RIK mice, and Dox-treated (5 days) 4E.389/CAGs-RIK mice that were then taken off Dox for two weeks (ON/OFF Dox). Bar represents 50 μm. (**D)** Representative immunohistochemistry staining showing mKate2 staining in the hair follicles of vehicle-treated (-Dox) and Dox-treated (+Dox) (5 days) 4E.389/CAGs-RIK or FLuc.1309/CAGs-RIK mice. Sections are from the experiment presented in Figure 1B. Bar represents 50 μm.

The generation of sh4E.389, sh4E.610 and shFLuc.1309 mice has been previously described [14]. CAGs-RIK mice harbor a CAGs promoter driving expression of rtTA3 and the fluorescent protein Kate2 targeted to the *Rosa26* locus (Figure 1A) (Dow, Nasr, Lowe, and Pelletier; In Preparation).

### Mouse studies

All mice strains were maintained on a C57BL/6 background. CAGs-RIK mice were crossed to sh4E.389, sh4E.610 and shFLuc.1309 mice [14] to generate bi-transgenic animals. Mice harboring the shFLuc.1309 allele serve as negative controls whereas using two independent sh4E alleles controls for off-target effects. Mice were genotyped by PCR amplification using the primers for CAGs-RIK (5′-GCTTGTTCTTCACGTGCCAG-3′ and 5′-CTGCTAACCATGTTCATGC-3′), sh4E.389 (5′-AATTACTAGACAACTGGATTGCCT-3′ and 5′-GAAGAACAATCAAGGGTCC-3′), sh4E.610 (5′-GCCACAGATGTATTTAGCTCTAAC-3′ and 5′-GAAGAACAATCAAGGGTCC-3′) and shFLuc.1309 (5′-CACCCTGAAAACTTTGCCCC-3′ and 5′-AAGCCACAGATGTATTAATCAGAGA-3′). All mice strains were maintained on a C57BL/6 background. shRNAmir activation was induced in mice by supplying doxycyline in the drinking water for the indicated periods of time. Dox-supplemented water was changed every 4 days.

### Cyclophosphamide (CyP)-induced alopecia

To synchronize hair growth in mice, hair was plucked from the back of mice. Nine days later (time of active hair growth at the anagen VI stage), mice were injected once with 150 mg/kg CyP by intra-peritoneal delivery. In experiments in which sheIF4E or shFLuc miRs were induced, Dox was added to the drinking water for 5 days prior to CyP delivery. Skin sections were harvested at days 12 and 21 post-depilation.

### Western blot analysis

For Western blot analysis, cells were lysed in RIPA buffer (50 mM Tris–HCl [pH 7.5], 150 mM NaCl, 1 mM DTT, 0.1% SDS, 1% NP-40, 0.5% sodium deoxycholate, 0.1 mM phenylmethylsulfonyl fluoride [PMSF], 1 mg/ml each of leupeptin, pepstatin, and aprotinin). Protein lysates were quantified by the Bio-Rad protein assay and 30 μg of proteins was resolved by SDS-PAGE, transferred to PVDF membranes (Millipore), probed with the indicated antibodies, and visualized using enhanced chemiluminescence (ECL) detection (Amersham). The antibodies used for protein expression analysis were directed against eIF4E (Cell Signaling, #9742), p53 (Santa Cruz, #sc-126), and tubulin (Sigma-Aldrich, #T5268).

### Ex Vivo treatment studies

Cells were cultured in triplicate in 6-well plates and pre-treated with 5 μM nutlin-3a, 40 nM hippuristanol, 40 nM Cr131-b, 10 μM 4E1RCat, or 10 μM 4E2RCat for 24 hours, followed by removal of the drug and exposure to 50 nM paclitaxel, 200 nM nocodazole, or 40 nM vinorelbine for 48 hours. The compounds were then removed and cells allowed to recover for 5 days. For eIF4E suppression, cells were transfected with siRNA against human eIF4E using Lipofectamine 2000 according to the manufacturer’s recommendations (Sigma-Aldrich). Two days later, cells were exposed to chemotherapy for 48 hrs, after which they were washed and allowed to recover for 5 days. Cells were counted using a Z2 Coulter Counter (Beckman Coulter).

For Giemsa staining, cells were fixed with ice-cold methanol-acetone (1:1 mixture) for 8 min at -20°C, and left to dry at RT. Giemsa solution (Sigma-Aldrich) was diluted 1:20 in PBS buffer and put on cells for 20 min at RT, after which time they were extensively washed with water. Plates were left to dry and visualized by microscopy (AxioScope; Zeiss).

For cell cycle analysis, cells (10^6^ cells/ml) were washed in PBS following compound treatment, fixed in 75% ethanol for 1 hour at 4°C, and stained with 50 mg/ml propidium iodide (Sigma-Aldrich) (containing 3.8 mM sodium citrate, and 500 mg/ml RNase A) for 3 hr at 4°C. DNA content was analyzed by FACScan (BD Biosciences).

### Immunohistochemistry analysis

Tissues were fixed in 10% neutral buffered formalin for 48 hours before embedding in paraffin and sectioned at 5 μm depth. Sections were dewaxed and rehydrated in a graded series of decreasing alcohol concentrations followed by a water wash. For antigen retrieval, sections were boiled for 15 min in 10 mM citric acid buffer (pH 6.0), followed by a 1 hr incubation in blocking buffer UltraVBlock (Anti-Rabbit HRP/DAB Detection Kit, Abcam), and a 10 min incubation with 3% hydrogen peroxide. Sections were then stained with rabbit primary antibodies against eIF4E (Cell Signaling, #9742, 1:50), GFP (Cell Signaling, # 2555, 1:800), mKate2 (Evrogen, #AB233, 1:800), and cyclin D1 (Cell Signaling, # 2926, 1:100) for 24 hours at 4°C, followed by incubation with biotinylated goat anti-rabbit IgG and streptavadin peroxidase (Anti-Rabbit HRP/DAB Detection Kit, Abcam) for 30 min each. Sections were washed with TBS buffer (0.1 M Tris–HCl (pH 7.5), 0.15 M NaCl) and the signal visualized using 3,3′-diaminobenzidine chromogen. Sections were counterstained with hematoxylin, dehydrated, and mounted using permount. Slides were scanned using an Aperio ScanScope (Aperio, Vista) and signals analyzed using an Aperio ImageScope (Aperio, Vista). Apoptosis was detected by TUNEL using the DeadEnd Fluorometric TUNEL System kit according to the manufacturer’s recommendations (Promega) and TUNEL positive cells were visualized using an Axio Observer fluorescent microscope (Zeiss).

### Statistics

For statistical analysis, unpaired Student t-test with Welch correction was performed using GraphPad InStat version 3.10.

### Study approval

All animal studies were approved by the McGill University Faculty of Medicine Animal Care Committee.

## Results

### Transient eIF4E suppression protects from CIA

In eukaryotes, modulation of eIF4E can lead to profound consequences on cell cycle progression [13-15]. We therefore sought to directly determine if suppression of eIF4E could protect against CIA. To this end, we took advantage of a recently developed transgenic mouse model in which we could potently suppress eIF4E in hair follicles in an inducible and reversible manner (Figure 1A, B) (Dow, Nasr, Lowe and Pelletier, In Preparation) [14]. As predicted, eIF4E was not suppressed in the hair follicle cells of FLuc.1309/CAGs-RIK mice - a control strain expressing a neutral shRNA to firefly luciferase [21] (Figure 1B). Importantly, eIF4E suppression could be reversed upon removal of doxycycline (Dox) from the drinking water (Figure 1C). Expression of Kate2 was used in all experiments as a surrogate marker to identify cells expressing rtTA3 (Figure 1D). These experiments highlight the value of CAGs-RIK mice in manipulating eIF4E levels in the hair follicle cells and in using Kate2 to track rtTA3 expression.

**Figure 2:**
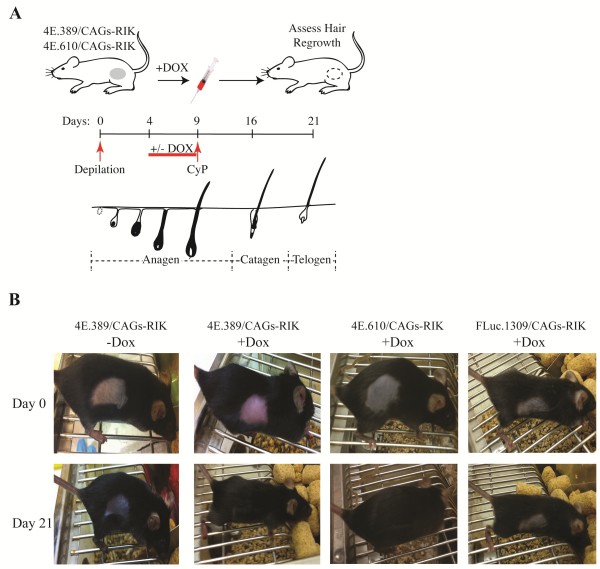
**Suppression of eIF4E protects from CIA. (A)** Schematic illustration showing experimental design for inducing shRNA expression and CIA in shRNA/CAGs-RIK mice. Shown are the timelines and the stages of hair growth induced upon depilation. In red is the time of Dox induction and CyP delivery schedule. **(B)** Mice of the indicated genotypes were depilated, exposed to Dox for 5 days prior to CyP treatment, and allowed to recover in the absence of Dox.

Hair growth in mice can be synchronized by depilation and proceeds through 3 stages – anagen (growth phase), catagen (regression phase), and telogen (resting phase) (Figure 2A). 4E.389/CAGs-RIK, 4E.610/CAGs-RIK and FLuc.1309/CAGs-RIK mice were depilated and following a four day recovery period were administered Dox or vehicle for 5 days followed by a single injection of CyP (at day 9 after depilation) (Figure 2B). Following recovery (with no Dox administered during this period) for 12 days, Dox-pretreated 4E.389/CAGs-RIK and 4E.610/CAGs-RIK mice showed full hair re-growth compared to Dox-pretreated FLuc.1309/CAGs-RIK or vehicle-treated mice (Figure 2B). These results indicate that suppression of eIF4E prior to chemotherapy delivery effectively protects against CIA.

**Figure 3:**
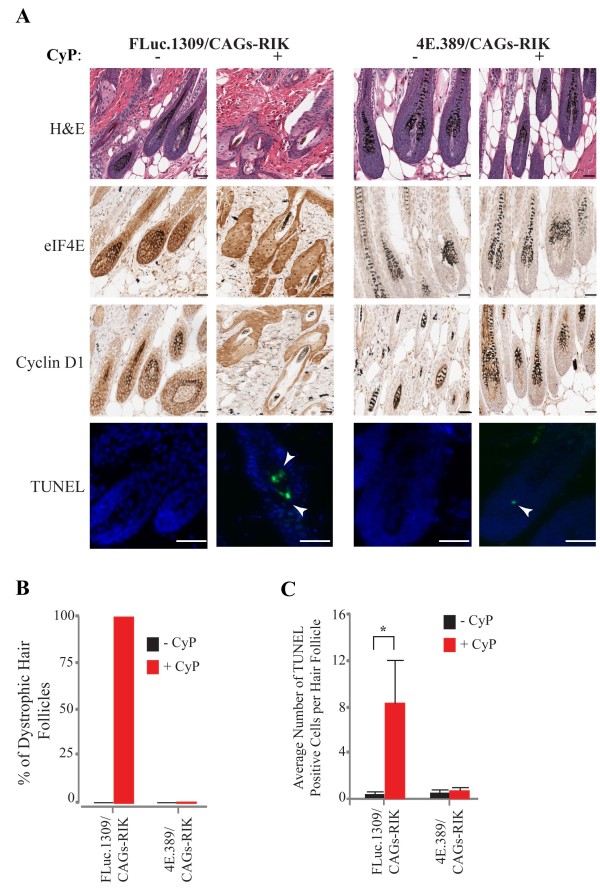
**Representative immunostaining of skin sections from Dox-treated shRNA/CAGs-RIK mice 3 days post-CyP. (A)** Skin sections from mice of the indicated genotypes were processed for H&E staining, immunostained for the indicated proteins, or processed for visualization of apoptotic bodies as described in the Methods. Bars = 25 μm. **(B)** Percent of dystrophic hair follicles in mice of the indicated genotype taken 3 days after cyclophosphamide delivery. n = 3 mice. Bars denote S.E.M. **(C)** Average number of apoptotic cells per hair follicle. Five different fields (5 follicles/field) were analyzed from sections obtained from 2 different mice. Bars denote S.E.M.

To better understand the consequences of eIF4E suppression on the hair follicles of CyP-treated mice, sections were prepared from skin harvested 3 days post-CyP treatment (Figure 3A). Dox-treated FLuc.1309/CAGs-RIK mice exposed to CyP showed dystrophy of the hair follicles, whereas Dox-treated 4E.389/CAGs-RIK mice exposed to CyP had follicles in the anagen phase - similar to mice that had not been exposed to CyP (Figure 3A, B; H&E stain). eIF4E levels were suppressed in sections of 4E.389/CAGs-RIK mice (Figure 3A; eIF4E) compared to FLuc.1309/CAGs-RIK mice, and this correlated with reduced expression of cyclin D1 (Figure 3A; cyclin D1), a known eIF4E-responsive target [22]. TUNEL staining revealed a significant proportion of apoptotic hair follicle cells in CyP-treated FLuc.1309/CAGs-RIK mice - as denoted by arrowheads (Figure 3A; TUNEL). In contrast, sections from CyP-treated 4E.389/CAGs-RIK mice in which eIF4E had been suppressed showed little evidence of apoptotic bodies (Figure 3A, C). These results demonstrate that eIF4E suppression prior to CyP treatment protects against CyP-induced apoptosis.

**Figure 4:**
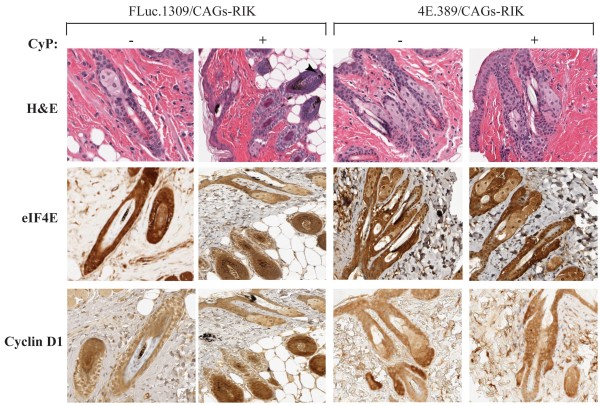
**Representative immunostaining of skin sections from Dox-treated shRNA/CAGs-RIK mice 12 days post-CyP exposure.** Representative immunostaining of skin sections from Dox-treated mice of the indicated genotype taken 12 days after cyclophosphamide delivery. Bars = 50 μm.

Histopathological examination of the hair follicles from Dox-treated FLuc.1309/CAGs-RIK mice, taken 12 days after CyP administration revealed that most were in the end of anagen/early catagen phases and showed remnants of disruption of melanin accumulation (intrafollicular/perifollicular ectopic melanin granules) (Figure 4), an indication of damage-response pathways of the hair follicles after chemotherapy [23]. In contrast, hair follicles of Dox-treated 4E.389/CAGs-RIK mice taken 12 days after CyP administration were in the final catagen or telogen stages (Figure 4), indicating that these follicles had transitioned through the entire growth cycle. At this stage, eIF4E and cyclin D1 expression had returned to normal levels in Dox-treated 4E.389/CAGs-RIK mice compared to Dox-treated FLuc.1309/CAGs-RIK mice (Figure 4).

### Suppression of eIF4E or eIF4A protects against chemotherapy induced cell death

**Figure 5:**
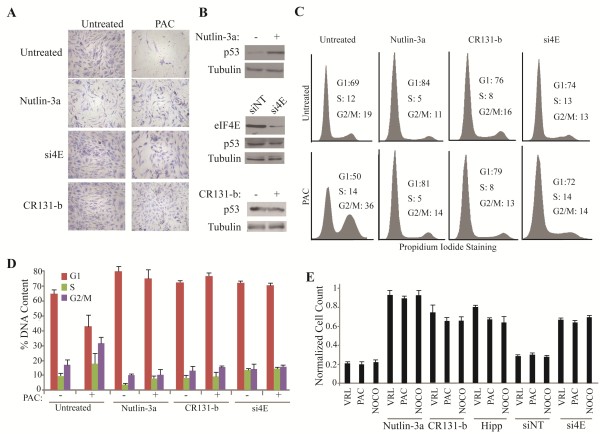
**Suppression of eIF4F protects against chemotherapy-induced cell death in non-transformed BJ/hTERT cells. (A)** Representative Giemsa staining of BJ/hTERT cells pre-treated with nutlin-3a or Cr131-b for 24 hrs, or transfected with siRNA against eIF4E (si4E) (2 days before treatment), followed by removal of compounds and exposure to paclitaxel (PAC) for 48 hrs. Cells were then allowed to recover for 5 days. **(B)** Western blot analysis of p53 and eIF4E from BJ/hTERT cells treated with nutlin-3a, Cr131-b or transfected with siRNA against eIF4E. **(C)** Representative cell cycle profiles of BJ/hTERT cells pre-treated as described in Panel A. **(D)** Quantification of the DNA content of BJ/hTERT cells pre-treated as indicated in Panel A. n = 3. Bars denote S.E.M. **(E)** Relative viability of BJ/hTERT cells that had been pre-treated with nutlin-3a, Cr131-b or hippuristanol (Hipp) for 24 hours or transfected with si4E or a non-targeting control (siNT) 2 days prior to the indicated drug treatments. Cell counts for VRL, NOCO, and PAC are normalized to controls in which cells were exposed to vehicle. n = 3. Bars denote S.E.M.

To better understand the molecular basis by which suppression of eIF4E leads to protection from chemotherapy-induced damage at the cellular level, we assessed the chemotherapeutic response of non-transformed cells as a consequence of eIF4E inhibition. As a positive control, exposure of hTERT-immortalized BJ cells to nutlin-3a afforded impressive protection to the mitotic poison paclitaxel (PAC) (Figure 5A). Suppression of eIF4E by RNA Interference (RNAi) afforded protection to PAC, as did inhibition of eIF4F activity using the small molecule inhibitor CR131-b (a rocaglamide previously referred to as (-)-9 in Ref [24]), which acts as a chemical inducer of dimerization and sequesters eIF4A from the eIF4F complex (Figure 5A) [25]. In these experiments, nutlin-3a induced p53 levels, whereas eIF4E suppression or CR131-b treatment did not, suggesting that the effects of eIF4E or eIF4A suppression on cell survival are not a consequence of p53 induction (Figure 5B).

**Figure 6:**
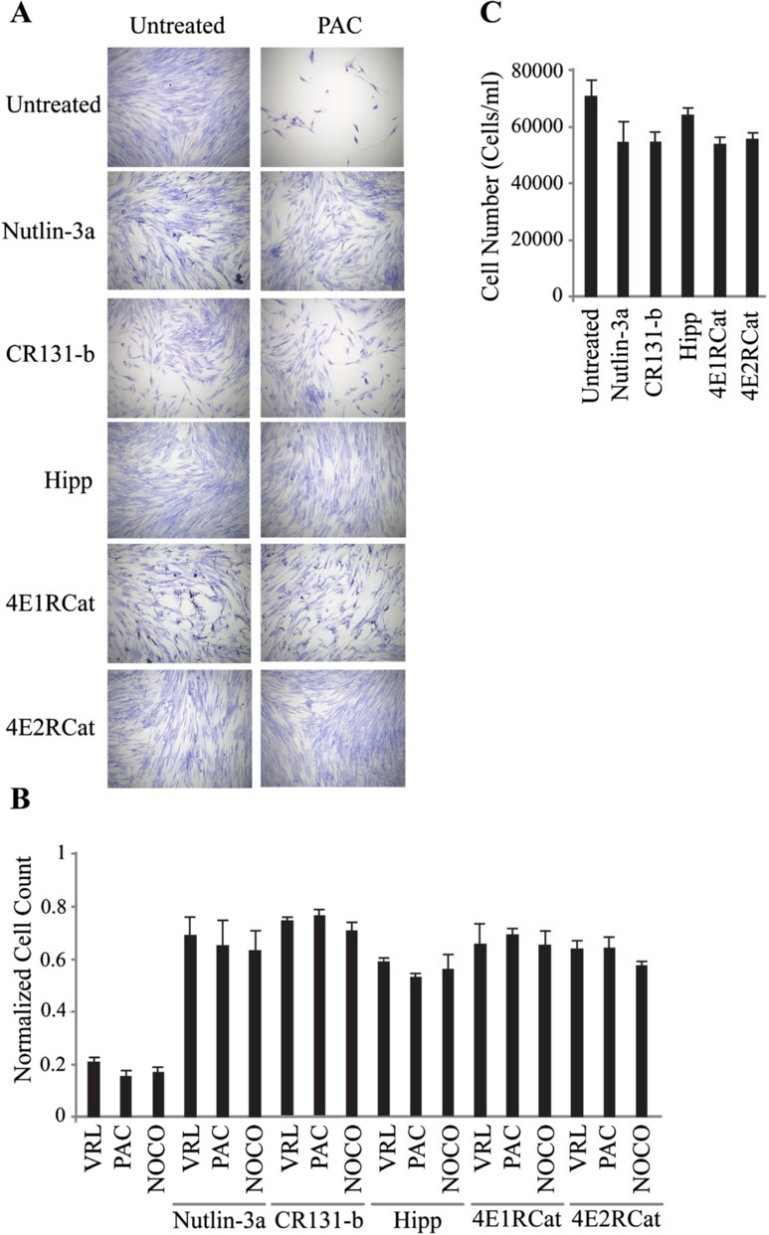
**Suppression of eIF4F protects against chemotherapy-induced cell death in non-transformed MRC5 cells. (A)** Representative Giemsa staining of MRC5 cells that had been pre-treated with nutlin-3a, Cr131-b, Hipp, 4E1RCat or 4E2RCat for 24 hours , followed by exposure to paclitaxel for 48 hrs. **(B)** Relative viability of MRC5 cells that had been pre-treated with nutlin-3a, Cr131-b, hippuristanol (Hipp), 4E1RCat or 4E2RCat for 24 hours and followed by exposure to 40 nM vinorelbine (VRL), 200 nM nocodazole (NOCO), or 50 nM paclitaxel (PAC) for 48 hrs. Cell counts for VRL, NOCO, and PAC were normalized to controls that had not been pre-treated. n = 3. Bars denote S.E.M. **(C)** Cell numbers after treatment of MRC5 cells with the indicated compounds for 24 hours and left to recover for 7 days. n = 3. Bars denote S.E.M.

We examined the cell cycle parameters of BJ/hTERT cells to characterize potential changes caused by the aforementioned treatments (Figure 5C-D). Exposure of BJ/hTERT cells to nutlin-3a or CR131-b, as well as RNAi-mediated suppression of eIF4E, caused an increase in the G1 population (Figure 5C-D). These results were not unique to PAC as these pre-treatments also protected from cell death induced by vinorelbine (VRL, a mitotic poison) and nocodazole (NOCO, a microtubule inhibitor) (Figure 5E and Additional file 1: Figure S1A). We also tested hippuristanol [26], an eIF4A inhibitor that has a completely different scaffold and mechanism of action compared to CR-131b, and obtained similar results (Figure 5E and Additional file 1: Figure S1A). As well, the eIF4E:eIF4G interaction inhibitors [27], 4E1RCat and 4E2RCat, provided protection from PAC, NOCO and VRL (Additional file 1: Figure S1A-C). These results were recapitulated in MRC5 cells, a non-transformed lung fibroblast cell line (Figure 6) indicating that the protective effects of blocking eIF4F activity are not cell line specific.

To determine if eIF4F activity had to be inhibited prior to drug treatment to obtain the observed protection, we treated BJ/hTERT cells with nutlin-3a, an eIF4A inhibitor (CR-131-b), or eIF4E:eIF4G interaction inhibitors (4E1RCat and 4E2RCat) concomitantly with PAC, NOCO, or VRL and noticed only a weak protection from cell death (Additional file 2: Figure S2). Taken together, these results indicate that suppression of eIF4E or eIF4A, prior to exposure of cells to cytotoxic agents, affords the greatest degree of protection to chemotherapy-induced cell death.

## Discussion

Alopecia is a frequent side effect of chemotherapy. Previous experiments of CIA in animal models have suggested the use of small molecule modifiers of the cell cycle to protect against chemotherapy. One example is the use of calcitriol (1,25-dihydroxyvitamin D3), known to induce G0/G1 arrest and inhibit DNA synthesis in keratinocytes [28]. Topical administration of calcitriol is able to protect from CIA in a neonatal rat model [29]. Although calcitriol did not fully protect adult mice from CIA, it facilitated hair re-growth by dampening CyP-induced apoptosis [30,31].

Using a novel transgenic model in which we could inhibit eIF4E expression using inducible shRNA technology, we demonstrated that eIF4E suppression *in vivo* afforded striking protection to CIA. We note that administration of the eIF4A inhibitor, CR131-b, by intra-venous injection to depilated mice for 5 consecutive days (once a day at 0.2 mg/kg) prior to CyP delivery failed to protect against CIA (data not shown). We attribute this to inadequate delivery of the compound to the intended target cells and these experiments will require more thorough knowledge of the tissue biodistribution and resident half-life of CR131-b in cells of the hair follicles, as well as appropriate surrogate markers to optimize the *in vivo* dose required to block cell cycling of the intended target cells.

Since inhibition of translation initiation by targeting eIF4F activity leads to accumulation of cells in G1 [14,32-34], it was reasonable to test the ability of several of the current translation initiation inhibitors in cyclotherapy. To date, several small molecules have been identified that either interfere with eIF4E-cap interaction, eIF4E:eIF4G interaction, or eIF4A helicase activity [17]. We showed that suppression of eIF4E, inhibition of the eIF4A helicase, or disruption of the eIF4E:eIF4G interaction provided significant protection to several chemotherapeutics *ex vivo* (Figures 5 and 6 and Additional file 1: Figure S1).

Suppression of eIF4E does not lead to global inhibition of protein synthesis but rather to a selective block in the ribosome recruitment phase of a subset of mRNAs. This would suggest that the expression of specific mRNA transcripts is affected in cells of the hair follicles and responsible for the cell cycle and apoptotic block. One potential mechanism is through reduced expression of cyclin D1, a key cell cycle regulator and known eIF4E target [22,35,36]. We postulate that the reduction of cyclin D1 in the hair follicles during anagen phase (Figure 3) blocks the majority of cells in G1, thus minimizing cell damage by CyP. This would be consistent with the reduction in apoptosis observed (Figure 3). We have not defined the eIF4E responsive mRNAs responsible for blunting CyP-induced apoptosis but this may simply be a consequence of the G1 block imposed by reductions in cyclin D1. Identifying such transcripts would require an unbiased and genome-wide approach to determining those mRNAs whose translation become altered during eIF4E suppression in the hair follicles. Overall, our results are in line with the principles of cyclotherapy [37,38].

We do not expect that eIF4E suppression or eIF4F inhibition will interfere with the efficacy of chemotherapy agents due to the absence of effective cell cycle checkpoints in cancer cells. Indeed, in many documented cases, the opposite is observed – that is, enhanced chemotherapy efficacy (synergy) in the presence of compounds that target translation [25,39-41]. Given that suppressing translation initiation appears a promising approach for cancer therapy, by using small molecule inhibitors of eIF4A or eIF4E:eIF4G interaction or using antisense oligonucleotides (ASOs) against eIF4E [17], the current results offer an added benefit of targeting translation for chemotherapy – that of protecting against CIA.

## Conclusions

In this study, we used a novel murine model that serves as a genetic approximation to drug target inhibition. Targeting the translation initiation factor, eIF4E, in non-transformed cells resulted in an accumulation of cells in G1, affording protection against chemotherapy-induced apoptosis. Suppression eIF4E in cells of the hair follicles provided profound protection against chemotherapy-induced alopecia. This correlated with a reduction in cyclin D1 levels and is consistent with a cyclotherapy response. Our results demonstrate the protective effect that inhibiting translation initiation has on minimizing CIA.

## Abbreviations

CIA: Chemotherapy-induced alopecia; eIF: Eukaryotic initiation factor; RNAi: RNA Interference; mTOR: Mammalian target of rapamycin; PI3K: Phosphoinositide 3-kinase; PMSF: Phenylmethylsulfonyl fluoride; FBS: Fetal bovine serum; P/S: Penicillin/Streptomycin; TBS: Tris-buffered saline; GFP: Green fluorescent protein; RMCE: Recombinase-mediated cassette exchange; shRNAs: Short hairpin RNAs; CAGS: Cytomegalovirus enhancer/chicken β-actin promoter; CAGs-RIK: Cytomegalovirus enhancer/chicken β-actin promoter–reverse tetracycline transactivator–internal ribosome entry site–Katushka 2; rtTA3: Reverse tetracycline transactivator 3; Kate2: Katushka 2; SDS: Sodium dodecyl sulfate; PAGE: Polyacrylamide gel electrophoresis; PVDF: Polyvinylidene fluoride; RT: Room temperature; PBS: Phosphate buffered saline; PCR: Polymerase chain reaction; CyP: Cyclophosphamide; Dox: Doxycycline; ECL: Enhanced chemiluminescence; hTERT: Human telomerase reverse transcriptase; PAC: Paclitaxel; VRL: Vinorelbine; NOCO: Nocodazole; Hipp: Hippuristanol; siNT: Non-targetting siRNA control; SEM: Standard error of the mean; TUNEL: Terminal deoxynucleotidyl transferase dUTP nick end labeling.

## Competing interests

SWL is founder on the scientific advisory board of Mirimus, Inc., a company that has licensed technology related to the mice used in this lab. All other authors declare they have no competing interests.

## Authors’ contributions

ZN performed genetic crosses, tissue preparation, immunohistochemical analysis of tissues, cell viability assays, cell cycle analysis, Western blot analysis, and drafted the manuscript. LED designed and characterized the CAGs-RIK mice. MP analyzed all tissue sections and provided expert advice on interpretation of results. JC performed immunohistochemical analysis of mouse tissues. KR, RS, and PD synthesized hippuristanol that was used in this study. JAP synthesized and provided CR-131b. SWL designed the CAGs-RIK mice and revised the manuscript. JP generated the CAGs-RIK mice, designed the study, drafted the manuscript, and revised the manuscript. All authors read and approved the final manuscript.

## Pre-publication history

The pre-publication history for this paper can be accessed here:

http://www.biomedcentral.com/2050-6511/14/58/prepub

## Supplementary Material

Additional file 1: Figure S1 Suppression of eIF4F protects against chemotherapy-induced cell death in non-transformed BJ/hTERT cells. **(A)** Cell count for BJ/hTERT cells treated with the indicated compounds or siRNAs for 24 hours and allowed to recover for 7 days. n = 3. Bars denote S.E.M. **(B)** Representative Giemsa staining of BJ/hTERT cells pre-treated with 4E1RCat and 4E2RCat for 24 hours followed by treatment with PAC for 48 hours and allowed to recover for 5 days. **(C)** Relative viability of BJ/hTERT cells that had been pre-treated with 4E1RCat or 4E2RCat for 24 hours followed by exposure to VRL, NOCO, or PAC for 48 hrs and allowed to recover for 5 days. Cell counts for VRL, NOCO, and PAC were normalized to cells exposure to vehicle. n = 3. Bars denote S.E.M. Click here for file

Additional file 2: Figure S2 Simultaneous inhibition of eIF4F with mitotic inhibitors does not protect against chemotherapy-induced cell death. **(A)** Representative Giemsa staining of BJ/hTERT cells treated with nutlin-3a, CR131-b, 4E1RCat or 4E2RCat in conjunction with paclitaxel for 48 hours. **(B)** Relative viability of BJ/hTERT cells that had been treated with nutlin-3a, Cr131-b, 4E1RCat or 4E2RCat and VRL, NOCO, or PAC for 48 hrs, then allowed to recover for 5 days. Cell counts for VRL, NOCO, and PAC were normalized to cells exposed to vehicle. n = 3. Bars denote S.E.M. Click here for file
