# Supplementary figures and images for: Suppression of eukaryotic initiation factor 4E prevents chemotherapy-induced alopecia
Source: BMC Pharmacol Toxicol. 2013 Nov 13;14:58. doi: 10.1186/2050-6511-14-58 (PMC4225821; doi:10.1186/2050-6511-14-58)

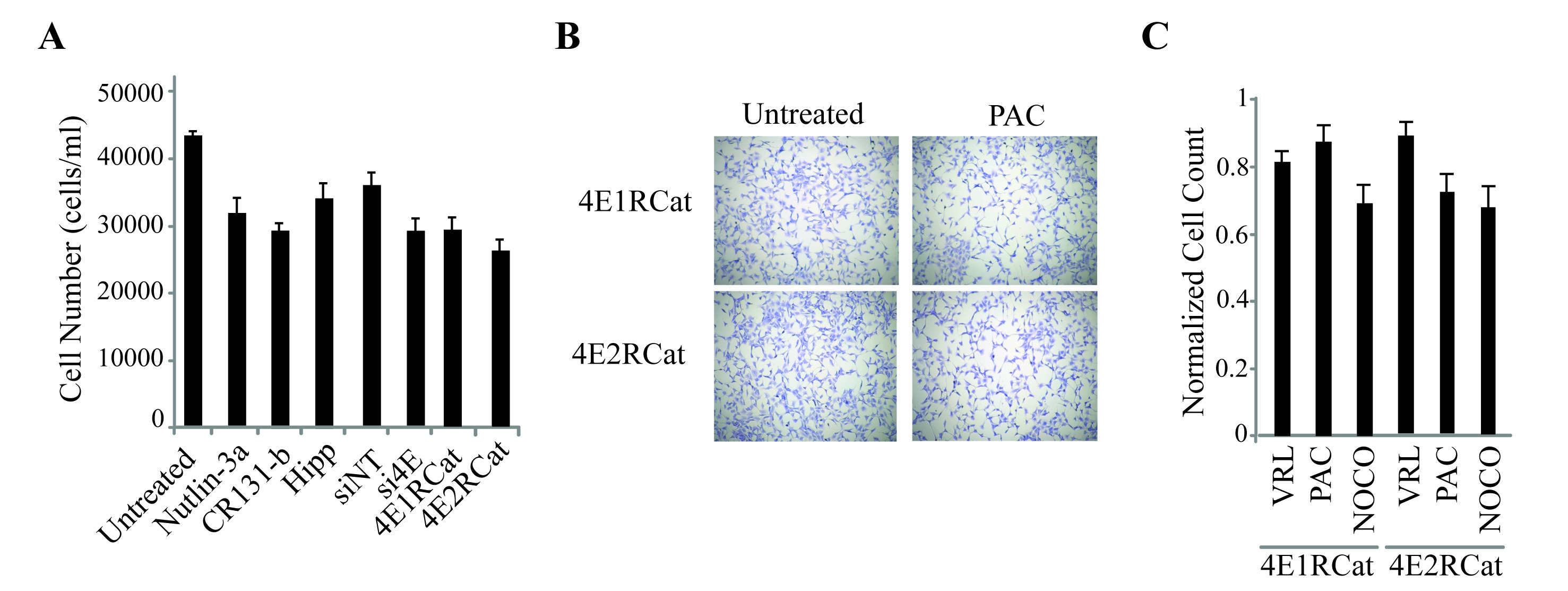

Supplement: Additional file 1: Figure S1 — Suppression of eIF4F protects against chemotherapy-induced cell death in non-transformed BJ/hTERT cells. (A) Cell count for BJ/hTERT cells treated with the indicated compounds or siRNAs for 24 hours and allowed to recover for 7 days. n = 3. Bars denote S.E.M. (B) Representative Giemsa staining of BJ/hTERT cells pre-treated with 4E1RCat and 4E2RCat for 24 hours followed by treatment with PAC for 48 hours and allowed to recover for 5 days. (C) Relative viability of BJ/hTERT cells that had been pre-treated with 4E1RCat or 4E2RCat for 24 hours followed by exposure to VRL, NOCO, or PAC for 48 hrs and allowed to recover for 5 days. Cell counts for VRL, NOCO, and PAC were normalized to cells exposure to vehicle. n = 3. Bars denote S.E.M. [file 2050-6511-14-58-S1.jpeg]

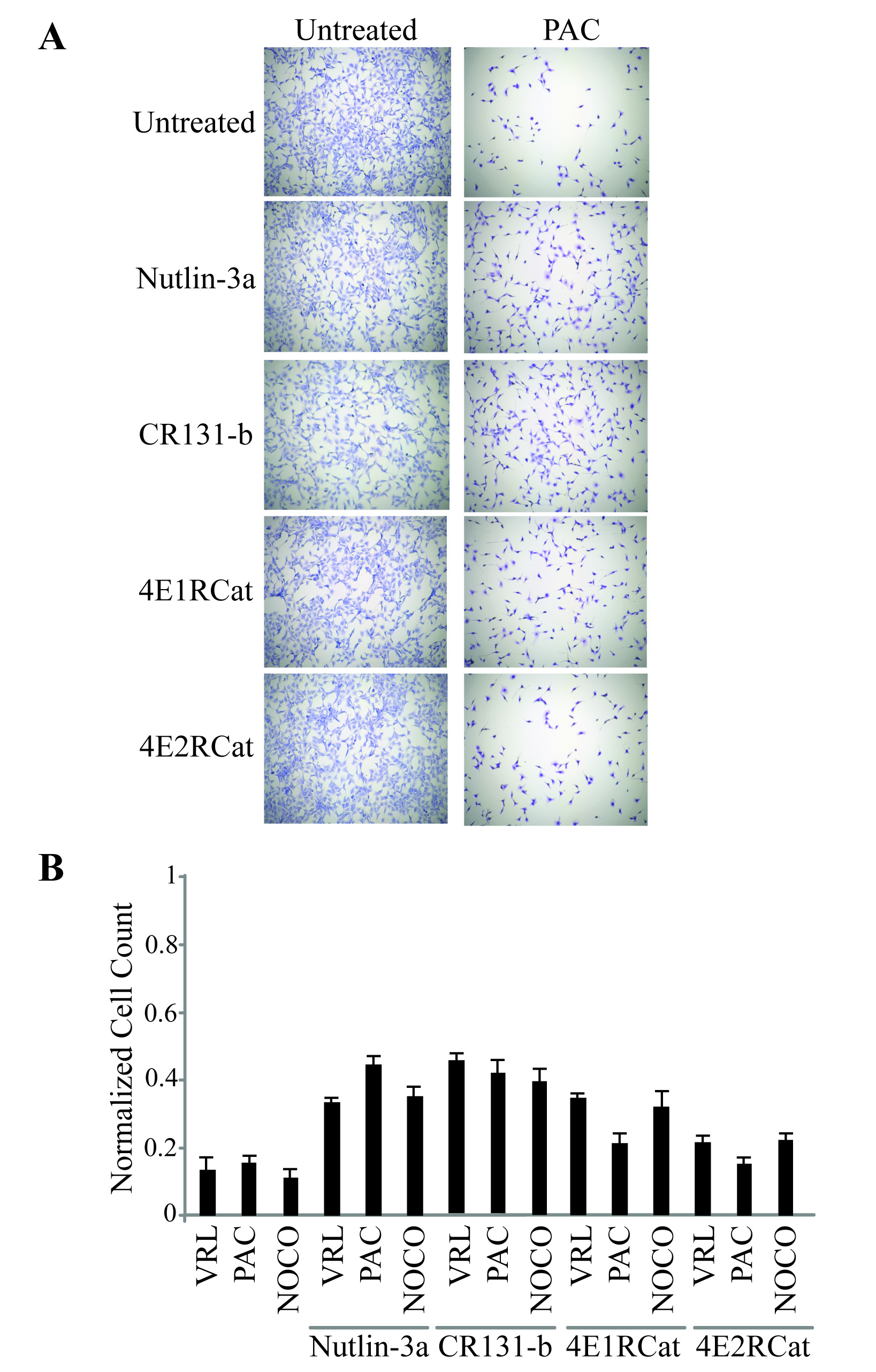

Supplement: Additional file 2: Figure S2 — Simultaneous inhibition of eIF4F with mitotic inhibitors does not protect against chemotherapy-induced cell death. (A) Representative Giemsa staining of BJ/hTERT cells treated with nutlin-3a, CR131-b, 4E1RCat or 4E2RCat in conjunction with paclitaxel for 48 hours. (B) Relative viability of BJ/hTERT cells that had been treated with nutlin-3a, Cr131-b, 4E1RCat or 4E2RCat and VRL, NOCO, or PAC for 48 hrs, then allowed to recover for 5 days. Cell counts for VRL, NOCO, and PAC were normalized to cells exposed to vehicle. n = 3. Bars denote S.E.M. [file 2050-6511-14-58-S2.jpeg]
